# Supplementary material for: Bridging genomics’ greatest challenge: The diversity gap
Source: Cell Genom. 2024 Dec 17;5(1):100724. doi: 10.1016/j.xgen.2024.100724 (PMC11770215; doi:10.1016/j.xgen.2024.100724)
Supplement: Document S1. Tables S1–S3 [file mmc1.pdf]

Cell Genomics, Volume *5*

Supplemental information

Bridging genomics' greatest challenge: The diversity gap

Manuel Corpas, Mkpouto Pius, Marie Poburennaya, Heinner Guio, Miriam Dwek, Shivashankar Nagaraj, Catalina Lopez-Correa, Alice Popejoy, and Segun Fatumo

Region	Census	Global % (8bn)	Year	Source
American	62,000,000	0.80%	2024	[1]
East Asian	1,661,506,318	20.8%	2024	[2]
European	1,280,000,000	16.0%	2019	[3]
Central/South Asian	2,047,895,034	25.6%	2024	[4]
Near Eastern	493,000,000	6.2%	2022	[5]
Oceanians	6,500,000	0.1%	2010	[6]
Sub Saharan African	1,210,000,000	15.1%	2022	[7]
Afro-American / Afro Caribbean	47,000,000	0.6%	2024	[8]
Latino*	701,548,639	8.8%	2024	[9] [10]

Supplementary Table 1: Data and sources from which we estimate global ancestry populations. Related to **Figure 4**.

* Summed up populations of Latin America + Latino population of US.

** There is some inevitable overlap between American and Latino populations.

Metaboliser status frequencies	American	East Asian	European	Central/South Asian	Near Eastern	Oceanian	Sub-Saharan African	African American/Afro-Caribbean	Latino
Ultrarapid metaboliser	5.1%	0.9%	2.3%	1.5%	7.4%	17.8%	3.6%	4.1%	4.1%
Normal metaboliser	64.9%	53.8%	49.2%	58.1%	56.5%	63.6%	25.4%	53.8%	59.6%
Intermediate metaboliser	23.1%	38.3%	38.3%	28.1%	30.1%	9.5%	33.9%	35.9%	29.1%
Poor metaboliser	2.0%	0.8%	6.5%	2.4%	2.2%	0.3%	2.0%	2.3%	3.1%
Indeterminate	4.9%	6.3%	3.7%	10.0%	3.7%	8.7%	35.0%	3.9%	4.2%

Supplementary Table 2: Predicted metaboliser phenotypes assigned to known PGx alleles by PharmGKB. Related to **Figure 6**. Source data from [11].

Predicted genetic ancestries	Approximate number of participants
Latino	1,312,000
African American	443,000
East Asian	314,000
West Asian and North African	82,000
South Asian	83,000
Other non-European	365,000

Supplementary Table 3. 23andMe's inference of genetic ancestry of participants within the ~10 million that have consented for research. Number of European participants is not provided by the source. Related to **Table 1**.

REFERENCES

1. Indigenous peoples of the Americas - Wikipedia https://en.wikipedia.org/wiki/Indigenous_peoples_of_the_Americas.

2. Population of Eastern Asia (2024) - Worldometer https://www.worldometers.info/world-population/eastern-asia-population/.

3. Genetics for all (2019). Nature Genetics 2019 51:4 *51*, 579–579. https://doi.org/10.1038/s41588-019-0394-y.

4. Population of Southern Asia (2024) - Worldometer https://www.worldometers.info/world-population/southern-asia-population/.

5. Demographics of the Middle East and North Africa - Wikipedia https://en.wikipedia.org/wiki/Demographics_of_the_Middle_East_and_North_Africa.

6. Oceania: Islands, Land, People | Cultural Survival https://www.culturalsurvival.org/publications/cultural-survival-quarterly/oceania-islands-land-people.

7. Sub-Saharan Africa - total population 2013-2023 | Statista https://www.statista.com/statistics/805605/total-population-sub-saharan-africa/.

8. African diaspora in the Americas - Wikipedia https://en.wikipedia.org/wiki/African_diaspora_in_the_Americas.

9. Latinos in the U.S. | Data on U.S. Hispanics | Pew Research Center https://www.pewresearch.org/race-and-ethnicity/fact-sheet/latinos-in-the-us-fact-sheet/.

10. Latino Countries 2024 https://worldpopulationreview.com/country-rankings/latino-countries.

11. Gene-specific Information Tables for CYP2D6 https://www.pharmgkb.org/page/cyp2d6RefMaterials.
